# Osseodensification versus piezoelectric internal sinus elevation (PISE) technique in delayed implant placement (a randomized controlled clinical trial)

**DOI:** 10.1186/s12903-024-04964-6

**Published:** 2024-10-28

**Authors:** Mohammed Samir, Mohamed Wagdy Bissar, Hala Ahmed Abuel-Ela

**Affiliations:** 1 AinShams University, Cairo, Egypt; 2 Periodontology and Oral diagnosis, Faculty of Dentistry, AinShams University, Cairo, Egypt

**Keywords:** Implant Dentistry, Postierior Maxilla, Sinus elevation, Osseodensification, Piezoelectric surgery, Sticky Bone, i-PRF

## Abstract

Transalveolar sinus elevation is a minimally invasive technique aimed at augmenting the vertical bone height in the posterior maxilla, facilitating successful implant placement in areas with insufficient bone volume. This study compares the efficacy of osseodensification and piezoelectric internal sinus elevation (PISE) techniques in delayed implant placement. The primary objective was to radiographically assess vertical bone gain and bone density, while secondary objectives included clinical assessment of primary implant stability and post-operative satisfaction of both patients and operators. The study population of a total of 16 patients was randomly divided into two groups. Group 1 underwent osseodensification sinus lift using sticky bone as a graft material, whereas Group 2 received PISE with the same graft material. Results indicated that the osseodensification technique led to greater bone gain, improved bone density, and shorter surgical duration. Additionally, osseodensification was associated with enhanced rapid healing and higher patient satisfaction. Conversely, the PISE technique demonstrated superior primary stability of implants on the day of surgery. These findings suggest that while both techniques are effective, osseodensification may offer advantages in terms of bone gain, density, and patient satisfaction, making it a reliable method for enhancing rapid healing in delayed implant placement. the study was registered on *clinicaltrials.gov* at 26^th^ September 2023 and clinical trials ID is *NCT06055127*.

## Introduction

Tooth loss is one of the major problems in restorative dentistry. Fixed and removable prostheses offer a solution for some cases but not all cases. Endosseous dental implants are an excellent solution for prosthetic reconstruction and rehabilitation for patients who lost their dentition mainly due to their predictability and long-term results [1–3]. However, like any treatment, dental implants have their limitations. One significant challenge is the limited bone quantity in both vertical and horizontal dimensions, particularly in the maxillary arch and more so in the posterior region. A primary solution to this issue is bone augmentation and regeneration, which includes techniques such as sinus elevation [4].

The posterior maxilla has distinct anatomical and physiological features that can make implant placement challenging. It is recognized as the most difficult and problematic area for implant dentistry, necessitating careful attention to ensure successful surgery. Some studies have reported a lower implant survival rate in the posterior maxilla, which is often attributed to the reduced bone density commonly found in this region [5].

This issue arises from alveolar bone atrophy and maxillary sinus pneumatization following tooth loss. Additionally, the maxilla’s poor bone quality, characterized by fine trabeculae with minimal or no cortical crest, makes it the least dense bone in the body. These factors can hinder the placement of sufficiently long dental implants, resulting in unsuccessful prosthesis loading and ultimately leading to implant failure [6].

The resorption pattern of the edentulous maxilla tends to be directed superiorly and medially. It has been demonstrated that the primary constraint for placing endosseous implants in the posterior maxilla is the height of the alveolar bone, rather than its width. Additionally, implant failures in the posterior maxilla without sinus lifting are often due to the use of implants that are too short to withstand the significant occlusal forces in this region, rather than the quality of type IV bone [7, 8].

To address the basic requirements for implant placement in the posterior maxilla, several techniques have been developed. Currently, two widely used methods for maxillary sinus augmentation are the Lateral Window Technique (LWT) and the Crestal Sinus Floor Elevation Technique (SFE). These techniques are recognized as two of the most reliable methods for achieving vertical augmentation in the oral cavity [9].

Many different bone grafting materials have been used in sinus augmentation to encourage or stimulate bone growth in this area.

Sticky bone is a fabricated growth factor-enriched bone graft matrix, using autologous fibrin rich blocks either with concentrated growth factors (CGF) or with liquid injectable platelet-rich fibrin matrices (I-PRF) [10].

Elevation of the Schneiderian membrane creates a compartment in which the blood clot is lodged. The stabilized blood clot has the potential to stimulate bone formation [11].

The treatment options for implant rehabilitation in the atrophic posterior maxilla can be broadly divided into two categories: a) Augmentation of the bony defect, which includes techniques such as grafts, guided bone regeneration, alveolar distraction osteogenesis, and sinus floor elevation. b) Modification of implant designs or positions, which involves placing implants in alternative anatomical regions, tilting implants, and using short implants [12].

Currently, the available sinus lift techniques used for implant placement in the pneumatized posterior maxillae are divided into two main methods:

1. The direct (open) method: lateral (window) sinus lifts either as a one or two-step procedure, direct sinus lift is also termed as lateral antrostomy or “Caldwell-Luc operation” [13, 14].
2. The indirect (closed) method: Internal sinus lift. The indirect sinus lift is also termed as crestal approach, subantral sinus augmentation, osteotome sinus floor elevation, “Summer’s technique”, subcrestal augmentation, sinus floor elevation or transalveolar approach [13, 14].

Indirect sinus lifting is a conservative, less invasive technique that is time-efficient and reduces patient morbidity. This method involves performing a small osteotomy through the crest of the edentulous ridge at the lower border of the antrum. This action elevates the Schneiderian membrane, creating a “tent” and providing space for the placement of graft biomaterial and the formation of a blood clot [15].

Multiple techniques were proposed to achieve safe and successful closed sinus lifting including:

### “Ultrasonic techniques”

Piezo-surgery is a surgical technique that was introduced by Italian oral surgeon ***Tomaso Vercellotti*** in 1988 to overcome the limits of traditional bone-cutting techniques such as diamond or carbide rotary instruments in oral bone surgery [16].

It uses ultrasonic waves that allow the ultrasound tips to oscillate and vibrate so that they can divide solid interfaces, such as bone tissue [17].

The piezoelectric device operates at an ultrasonic frequency typically between 25 and 30 kHz, allowing it to selectively cut mineralized tissues while preserving soft tissues like the Schneiderian membrane or nerve tissue (which require a frequency of 60 kHz for cutting). This frequency generates micro-vibrations with an amplitude of 60-210 μm and a power range of 2.8 to 16 watts. This selective cutting capability makes the procedure safer for accessing the sinus, with minimal risk of membrane injury or perforation [18].

### Maxillary sinus floor augmentation through bone densification:

Osseodensification is a novel technique, wherein highspeed densifying burs are used in increasing sizes to preserve and compact the bone as the sinus floor is being elevated [19]. The use of Densah burs for preparing implant site had many advantages including the increase of implant bone contact by compaction autografting rather than excavation of bone in conventional drill, this mainly depends on the viscoelastic nature of bone where time dependent stress produces time dependent strain, it also allows for higher insertion torque and increased stability of dental implant [19]***.***

The osseodensification effect is due to the drill design. It presents many faces and a negative cutting angle, possibly increasing bone density while expanding the bone tissue during osteotomy [20]. Thus, these drills' design promotes compaction of the bone tissue, increasing its density laterally and, apically, improving the initial stability of the implant [20–23]. This fact can be observed in preclinical and clinical studies, which showed favorable results after applying the technique [24, 25].

It is a recent innovation that has the added benefits of preserving bone during osteotomy preparation, increasing the primary stability of the implant, which in turn helps in better osseointegration and success of the implant [26].

The alveolar crest Osseodensification sinus lift technique is a conservative, minimally invasive, and low-trauma procedure. It utilizes hydro-pneumatic, counterclockwise rotating instruments to densify bone and elevate the maxillary sinus floor without contacting the Schneiderian membrane, thereby minimizing the risk of perforation [27, 28].

Sticky bone is a fabricated growth factor-enriched bone graft matrix, using autologous fibrin rich blocks either with concentrated growth factors (CGF) [29] or with liquid injectable platelet-rich fibrin matrices (I-PRF).[30] granting the stabilization of the bone graft in bony defects which minimizes bone loss during healing. Furthermore, promoting healing by the significant release of cytokines and autologous growth factors [31, 32].

Sticky bone, a third-generation platelet concentrate, is rich in various growth factors such as VEGF, PDGF, IGF, EGF, FGF, TGF-β, and BMPs. BMPs can independently mediate osteogenesis or, when combined with other growth factors, enhance the development and calcification of the bone matrix [33].

TGF-β is a key regulator of the formation and remodeling of bone. It can stimulate the regeneration of alveolar bone and control inflammation by stimulating the synthesis of fibrous connective tissues and local vascular proliferation [33].

Nonetheless, the easy handling of the grafting material reduces the surgical time. All these factors indicate that sticky bone is a promising autologous bone graft material for bone tissue regeneration [34].

Up till now there are limited studies to evaluate the transcrestal sinus lift and simultaneous implant placement using osseodensification and piezoelectric internal sinus elevation (PISE) technique, Hence, this study was planned to compare osseodensification versus piezoelectric internal sinus elevation (PISE) technique in delayed implant placement with placement of sticky bone as bone grafting material. The aim of the present study is to evaluate the effectiveness and clinical results of Osseodensification in comparison to Piezoelectric Internal Sinus Elevation (PISE) Technique in Delayed Implant Placement.

- Primary objective: Radiographic assessment of vertical bone gain and bone density.
- Secondary objective: Clinical assessment of primary stability of the implants.
- Secondary objective: Assessment of patient and operator satisfaction post-operatively.

### Subjects and methods

For the sample size calculation, a power analysis was designed to have adequate power to apply a two-sided statistical test of the null hypothesis that there is no difference would be found between tested groups. By adopting an alpha (α) level of (0.05), a beta (β) of (0.2) (i.e., power=80%), and an effect size (d) of (1.63) calculated based on the results of a previous study [35]. The minimal required sample size (n) was found to be (14) cases. Sample size was increased by (20%) to compensate for possible dropouts during follow-up intervals to be (16) cases (i.e., 8 cases per group). Sample size calculation was performed using G*Power version 3.1.9.7.

The present study was a parallel single-blinded randomized controlled clinical trial with a total of sixteen patients, selected from the outpatient clinic of Oral Medicine, Periodontology and Oral Diagnosis Department, Faculty of Dentistry, Ain Shams in Egypt.

Ethics approval and consent to participate’: The study followed the protocols of the Declaration of Helsinki and was conducted after being reviewed and approved by the Faculty of Dentistry, AinShams University Research Ethics Committee (FDASU-REC) (approval code: *FDASU_Rec IM 122215)*

The study was registered on *clinicaltrials.gov* at 26^th^ September 2023 and clinical trials ID is *NCT06055127 (*https://clinicaltrials.gov/study/NCT06055127*) and* adheres to CONSORT guidelines for reporting clinical trials

A comprehensive explanation of the surgical procedure was given to all the participants including the possible risks and alternative prosthetic solutions. Each participant signed a detailed written patient consent.

The study population was randomly allocated into two groups of the same size:

Group 1:

Osseodensification sinus lift was performed using sticky bone as a graft material.

Group 2:

Piezoelectric Internal Sinus Elevation (PISE) was performed using sticky bone as a graft material.

The sample randomization was obtained by the aid of a computer-generated randomization table before the start of the surgical procedures.

Inclusion criteria:

1. Patient partially edentulous with maxillary posterior edentulous ridge after extraction of more than 4 months.
2. Both genders will be selected males and females.
3. Adult patients aged between 18 and 40 years of age.
4. Good general health (American Society of Anesthesiology Physical Status I-II).
5. Initial residual alveolar ridge height ranging between 4 to 6 mm according to preoperative CBCT.
6. No previous surgery or radiation treatment on the maxillary sinus [35].

Exclusion criteria:

1. Smokers.
2. Pregnant or lactating females.
3. Psychiatric disorders.
4. Uncontrolled systemic disease.
5. Hematologic diseases and coagulation disorders.
6. Chemotherapy or radiotherapy of the head and neck area, and immune-compromised status.
7. Medical conditions affecting bone metabolism and ongoing treatment with bisphosphonates drugs or systemic steroids.
8. Presence of acute or chronic sinus pathoses or sinus membrane perforation [35].

### Pre-surgical procedure

A comprehensive explanation of the surgical procedure was given to all the participants including the possible risks and alternative prosthetic solutions. Each participant signed a detailed written patient consent.

Cone Beam Computed Tomography *(CBCT i-CAT next-generation scanner(Imaging Sciences International,Hatfield,PA,USA) operating at a tube voltage of 120KVp,tube current of 5mA,voxel size of 0.2mm,field-of-view of 16x6cm ,and scanning time of 26.9seconds)* was made to assess the sinus anatomy , the height and width of the residual ridge from the sinus floor to the alveolar crest of each participant and take an estimate measurement of bone density with Hounsfield units (HU).

- Pre-surgical medication consisted of intravenous steroidal anti-inflammatory drug (dexamethasone sodium phosphate 4mg) one hour before the procedure.
- Oral rinsing with 0.12% chlorhexidine for 1 minute.

### Surgical phase

**Fig. 1:**
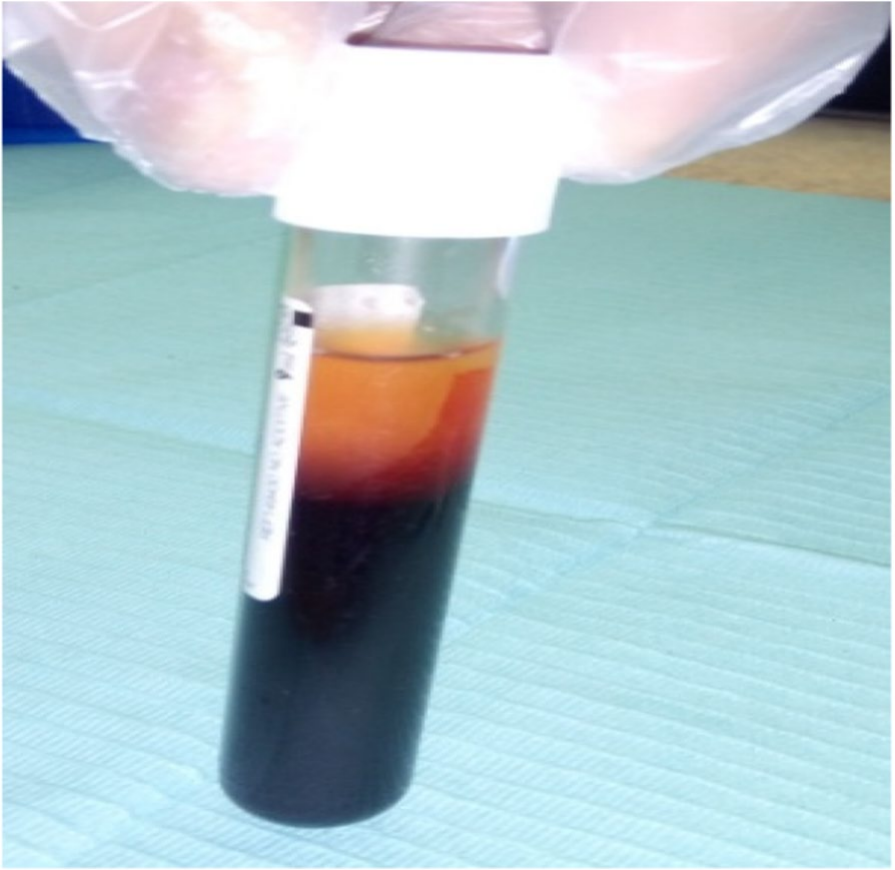
10 ml whole blood was drawn into glass-coated plastic tubes without anticoagulant and centrifuged immediately at 700 rpm for 3 minutes

**Fig. 2:**
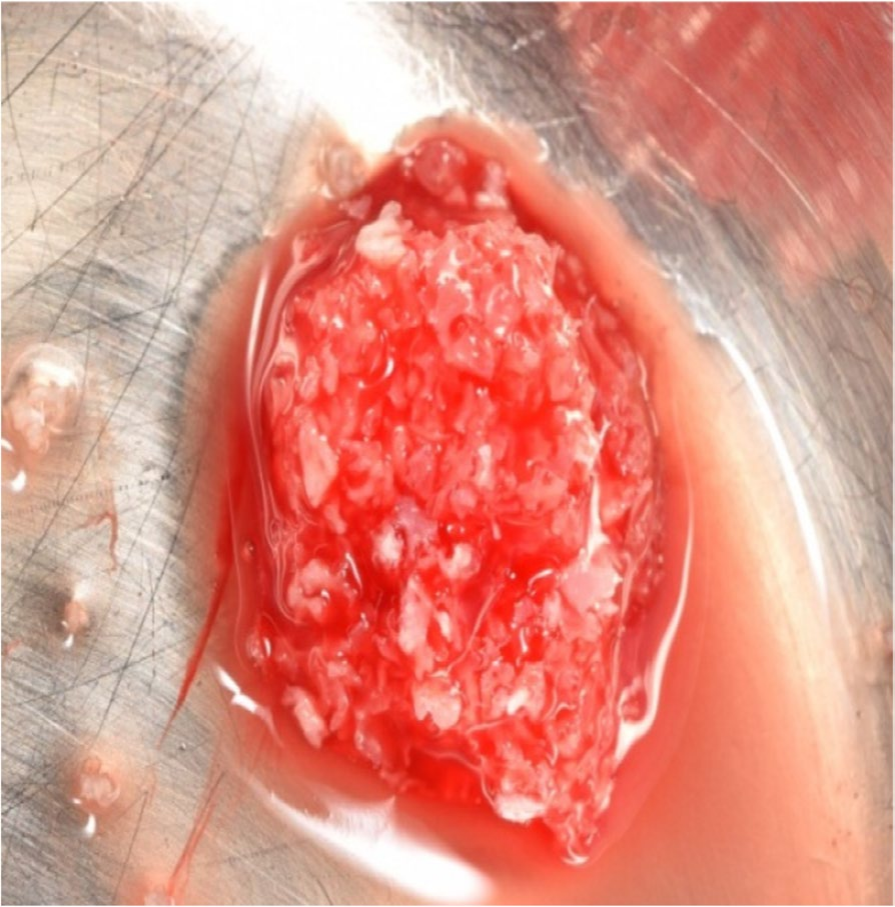
Red colored sticky bone

Sticky bone was prepared as described by (*Mourão*) [30], 20 ml whole blood was drawn into two glass- coated plastic tubes without anticoagulant and centrifuged immediately at 700 rpm for 3 minutes (Fig. 1). The 1 mL upper plasma layer was collected using a 20-gauge needle and named i-PRF. Addition of i-PRF to particulate bone will lead to polymerization in 15 minutes to produce red colored sticky bone (Fig. 2).

Local anesthesia consisting of articaine hydrochloride 4% and epinephrine1:100000. Crestal and vertical releasing incisions were made, a full thickness mucoperiosteal flap was lifted and a maxillary alveolar process was revealed.

### In Group 1

**Fig. 3:**
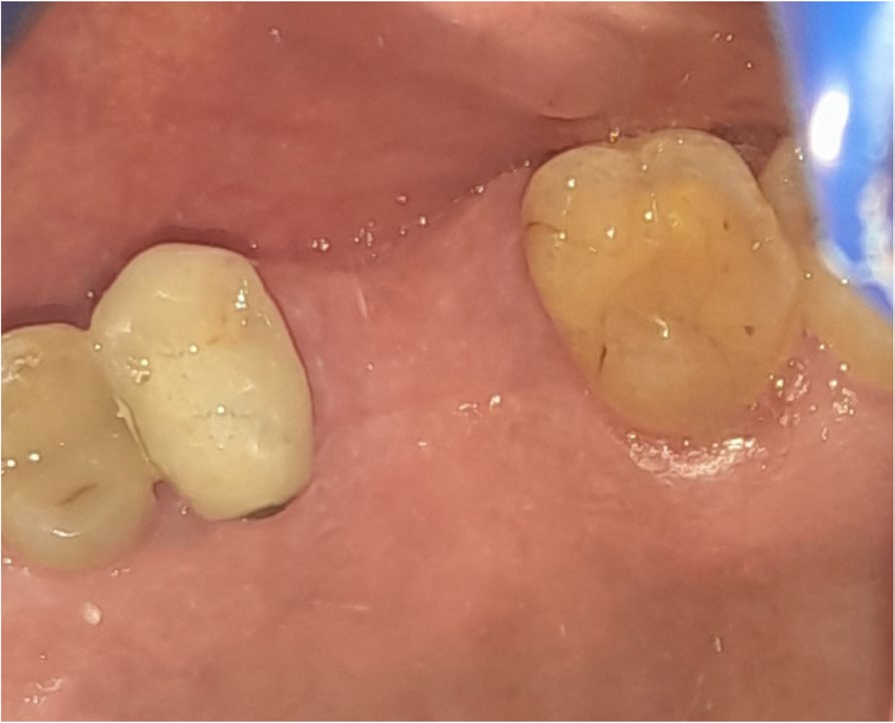
Pre-operative clinical occlusal view (missing Upper 1^st^ molar)

**Fig. 4:**
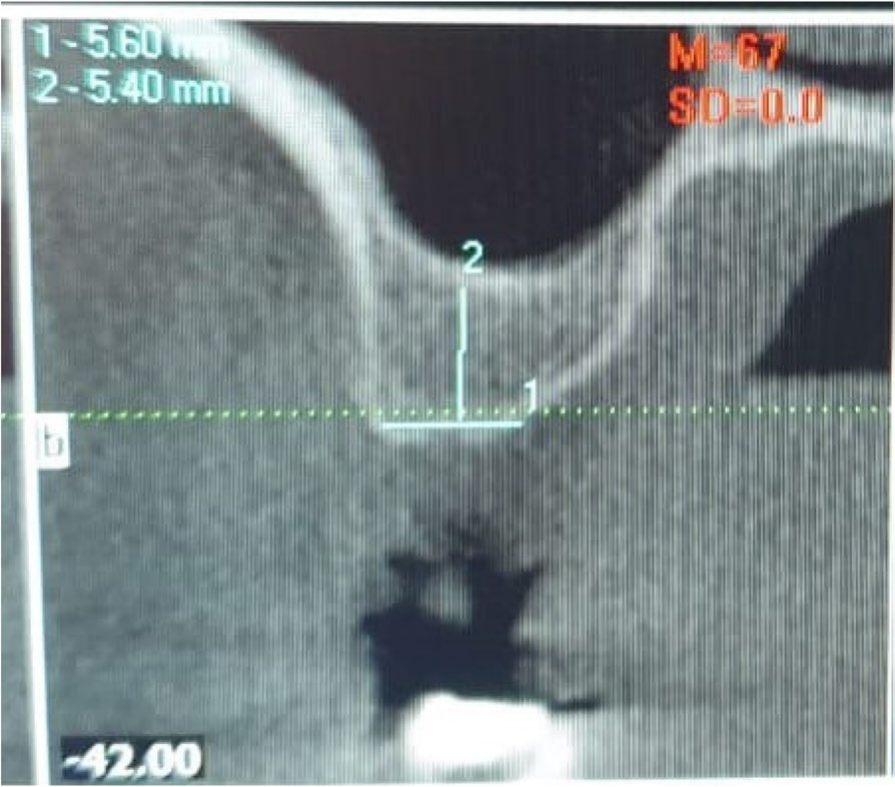
Per-operative CBCT cross-section cut

**Fig. 5:**
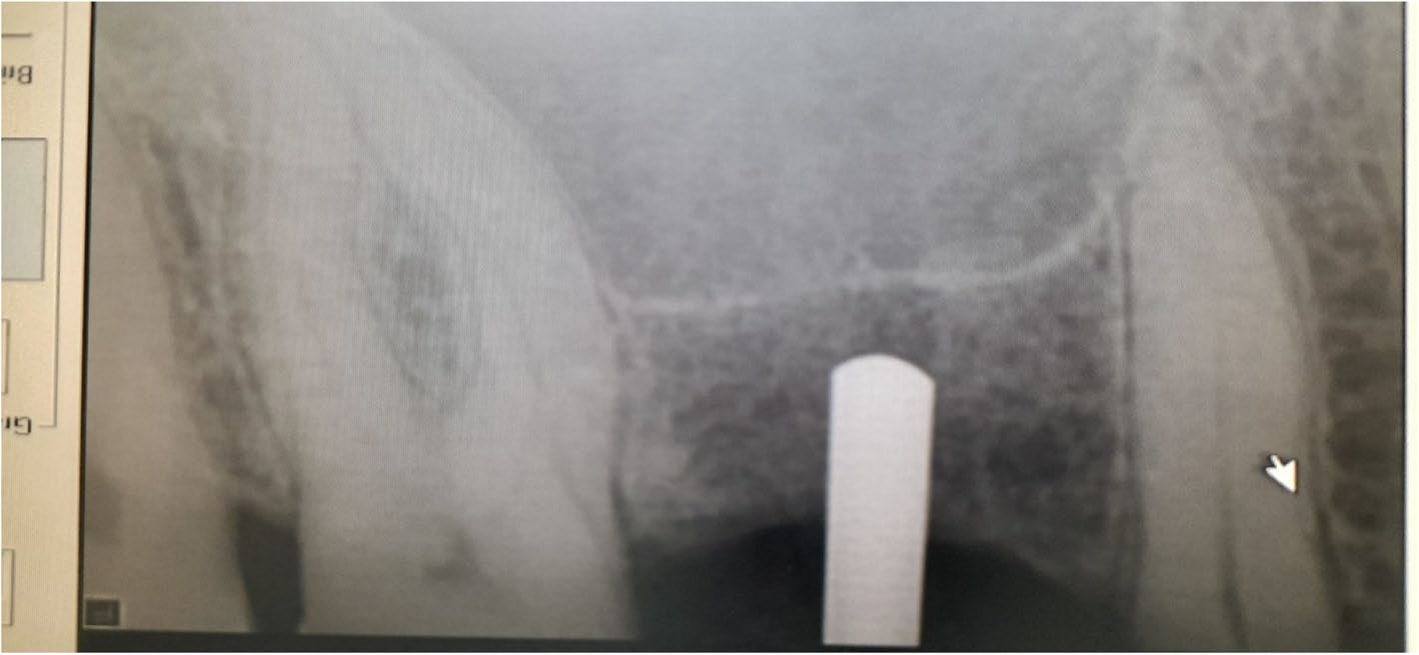
Paralleling pin 2mm from the sinus floor

**Fig. 6:**
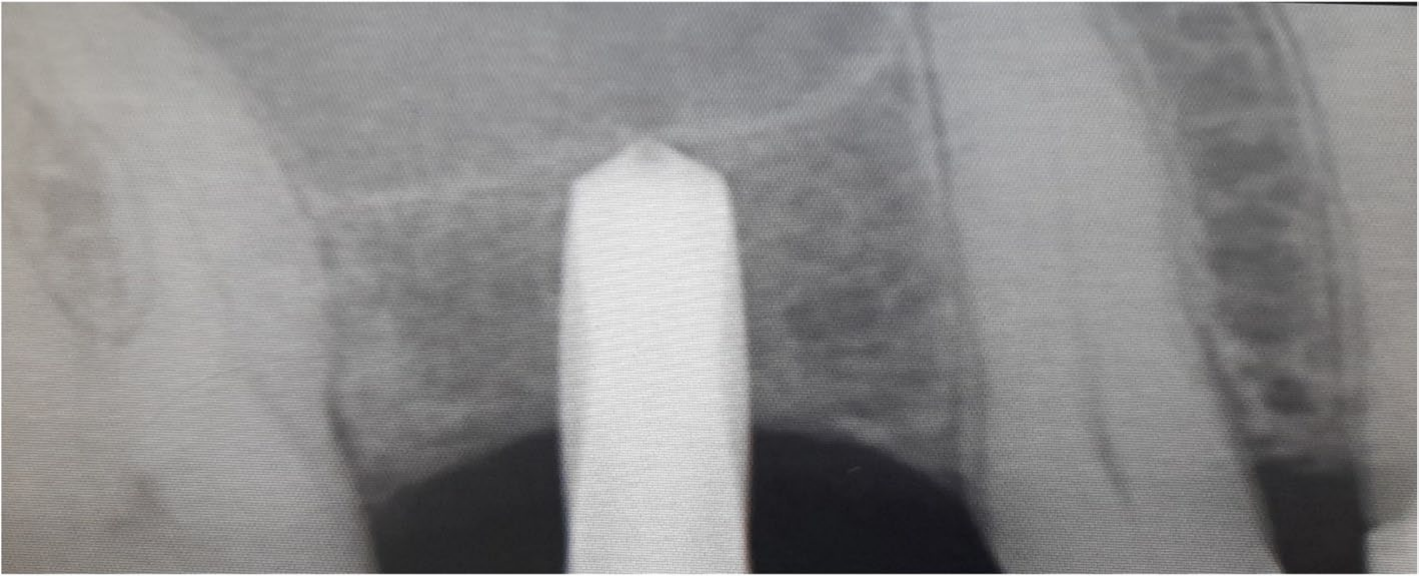
Final osteotomy drill was advanced with gentle pressure at 100 rpm counterclockwise without irrigation until a bouncing sensation occurs “haptic feedback” at this point infracture of the sinus had taken place

**Fig. 7:**
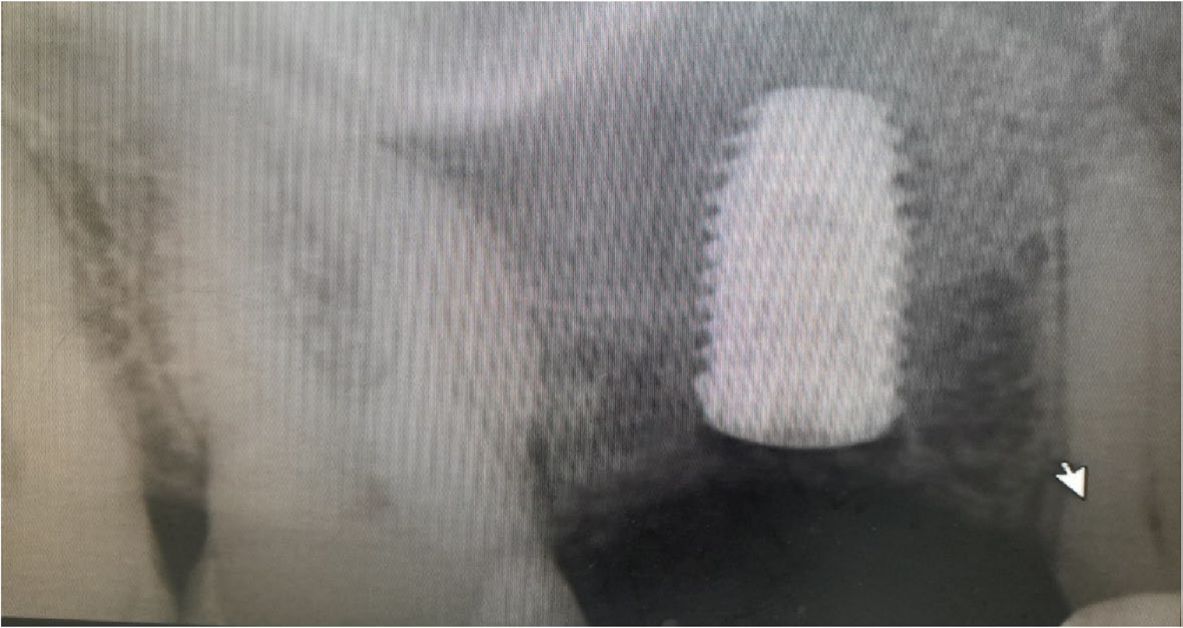
Post-operative periapical Radiograph of implant in place after sinus floor elevation

**Fig. 8:**
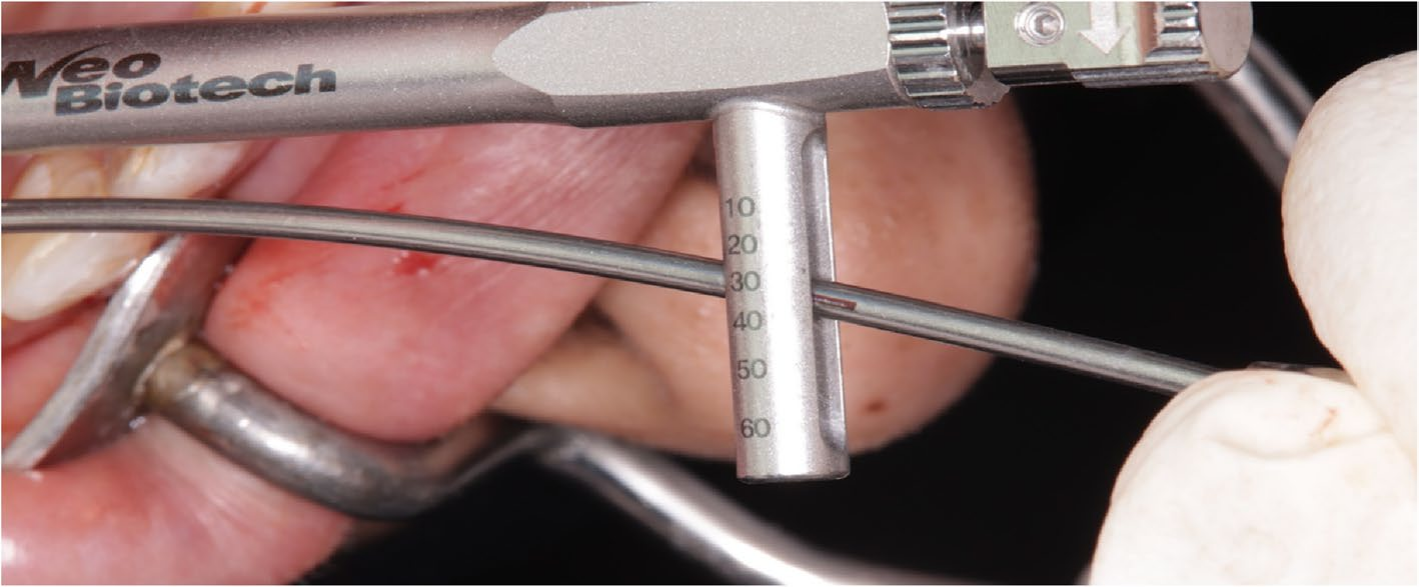
Torque wrench (measuring 30-35N/cm^2^)

**Fig. 9:**
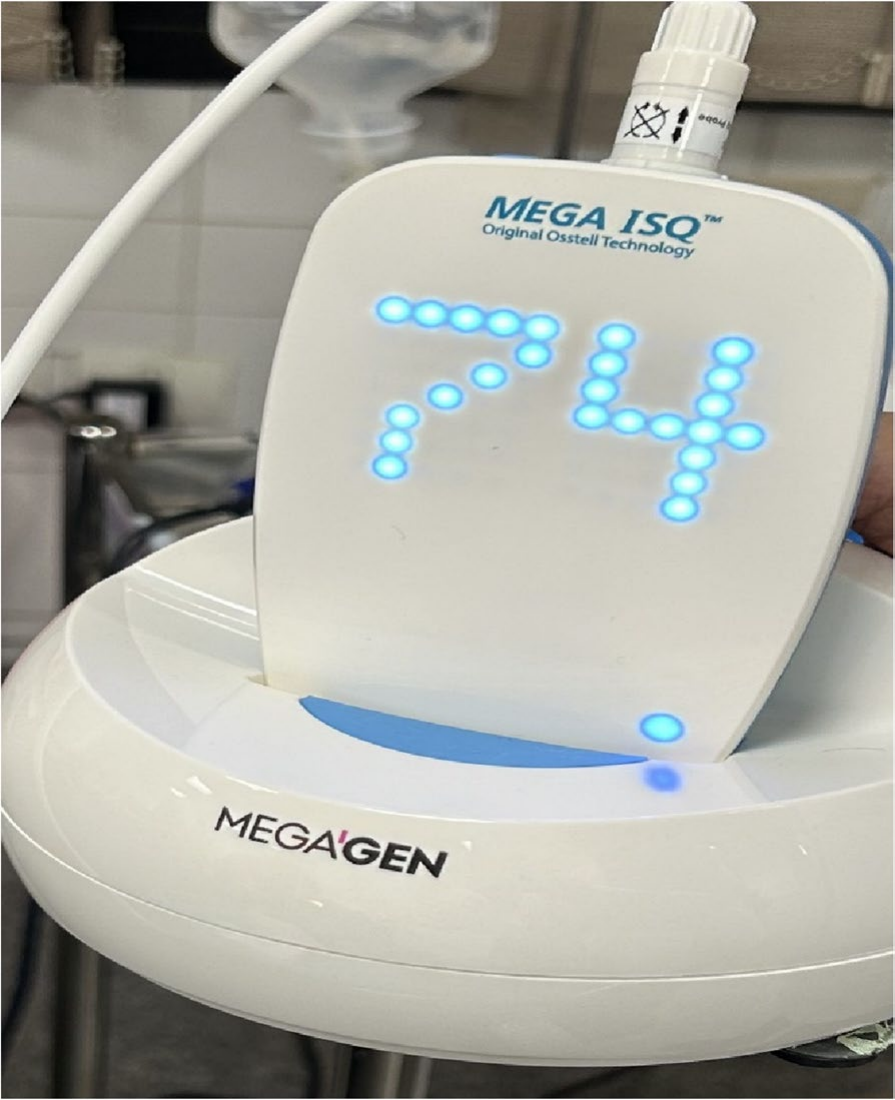
ISQ value

**Fig. 10:**
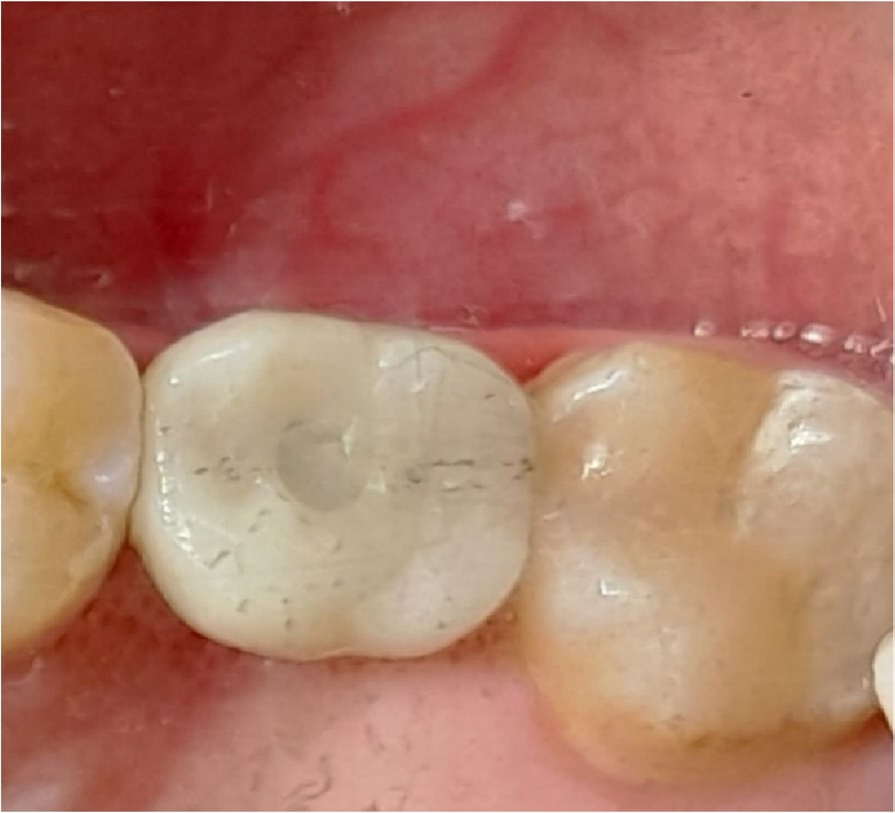
Final screw retained crown after implant osseointegration

The osteotomy for the osseodensification internal sinus lift is made by Versah LLC. Universal Densah® Bur Kit [36]. The osteotomy begins with a twist drill advanced at 800 rpm with saline irrigation to within 1-2 mm from the sinus floor (Figs. 3, 4 and 5). Next, a series of osteotomy drills were used in the same fashion at 800 rpm to widen the osteotomy. The final osteotomy drill was advanced with gentle pressure at 100 rpm counterclockwise without irrigation until a bouncing sensation occurs “haptic feedback” at this point infracture of the sinus had taken place (Fig. 6). After infracture, injection of the graft material into the osteotomy site was done. The final osteotomy drill was used to guide the graft apically. This process was repeated in an incremental fashion to lift the membrane. Once enough space has been created below the antral membrane, implant insertion was done (Fig. 7), insertion torque was measured by torque wrench (Fig. 8) and ISQ was measured using an Osstell ISQ Scale (Figs. 9 and 10) [37] then suturing was done.

### In Group 2

**Fig. 11:**
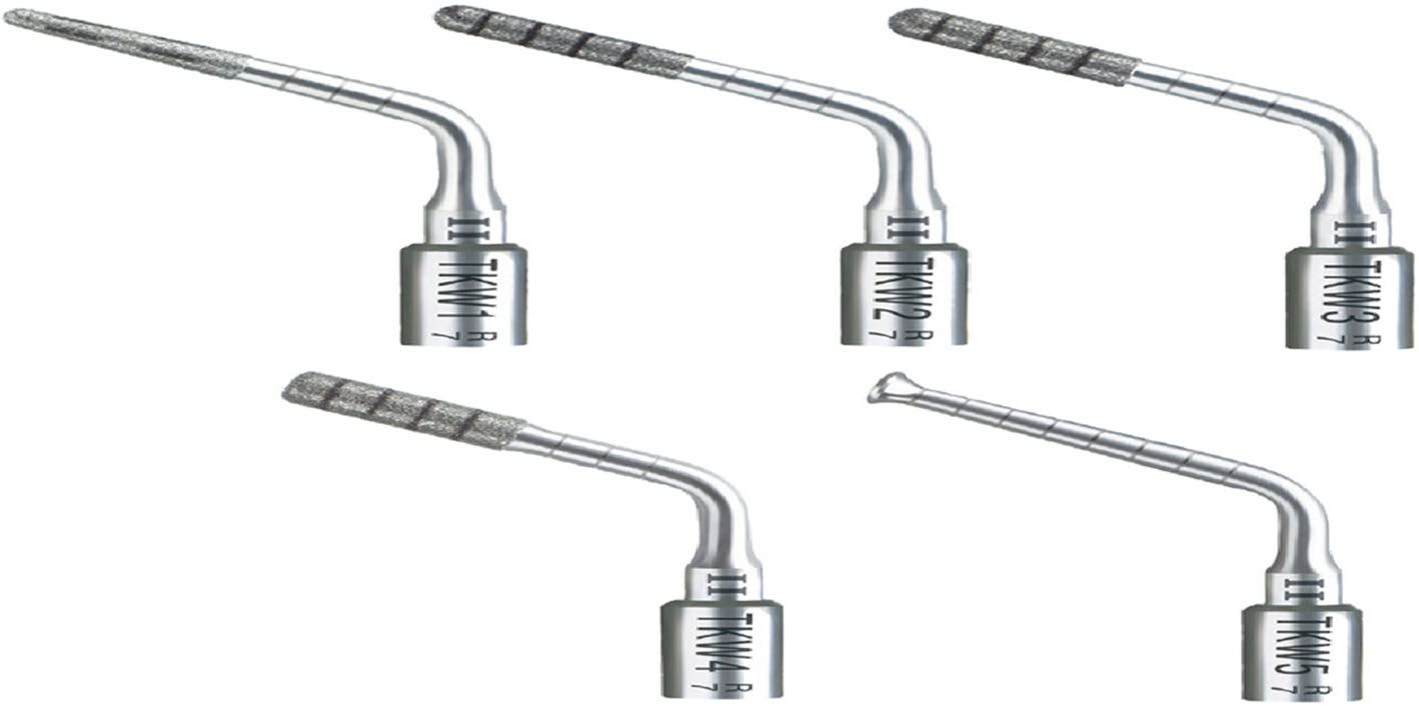
Intralift™ Kit

**Fig. 12:**
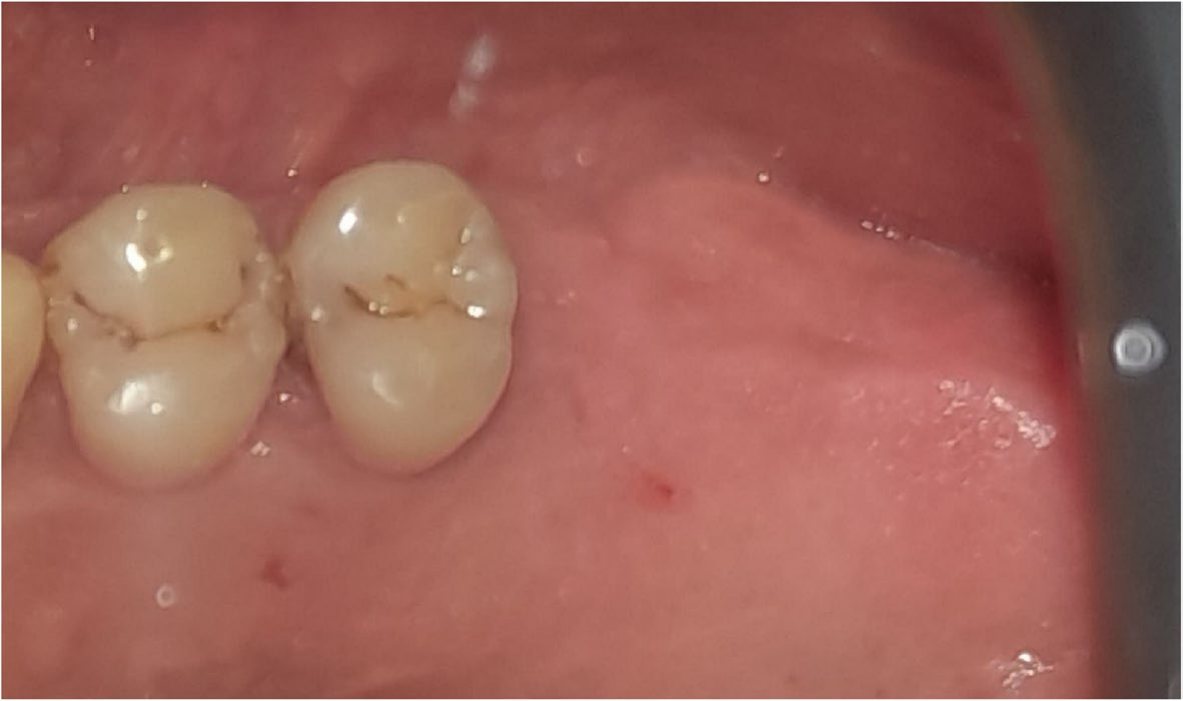
Pre-operative clinical occlusal view (missing Upper 1^st^ molar)

**Fig. 13:**
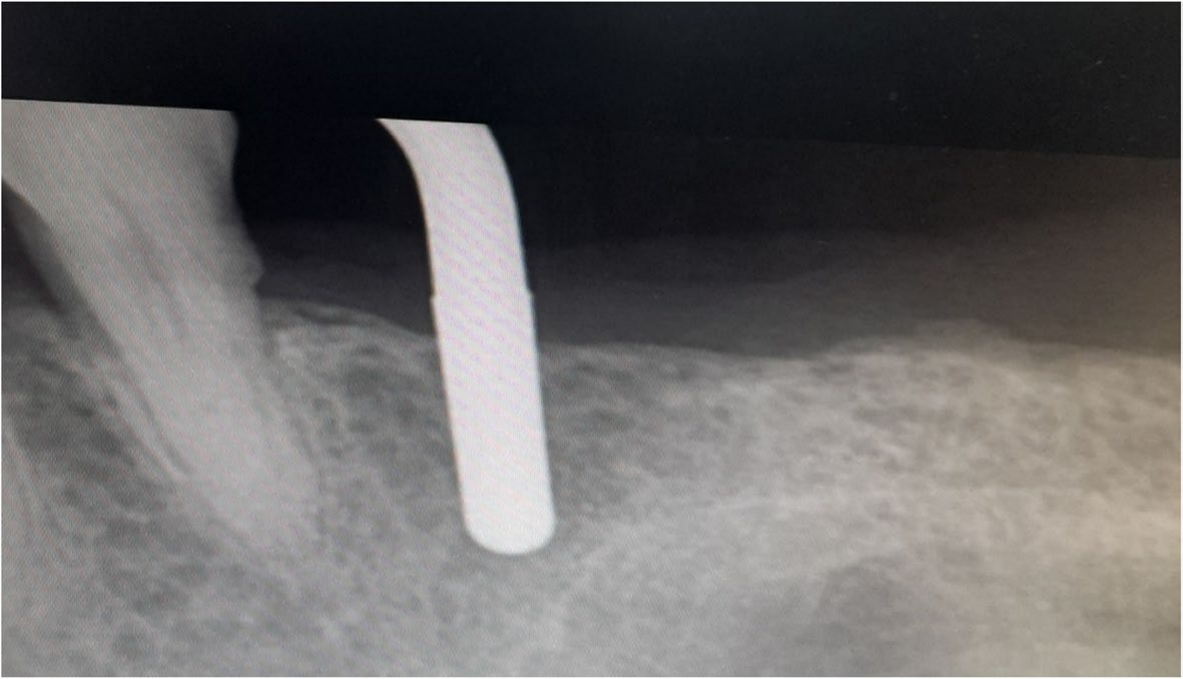
TKW 3 advanced to 1-2 mm from the sinus floor

**Fig. 14:**
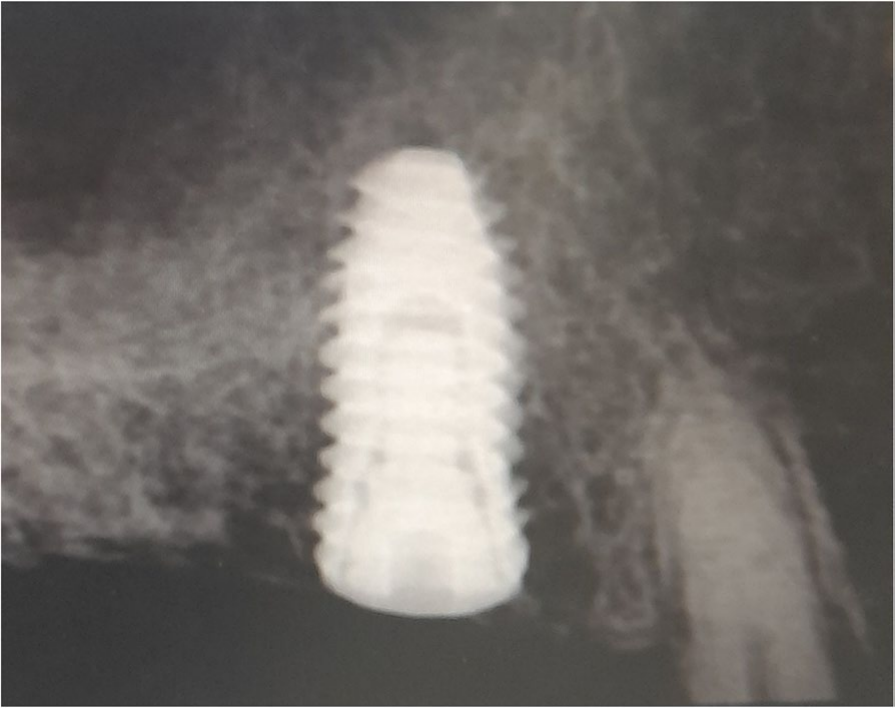
Post-operative periapical Radiograph of implant in place after sinus floor elevation

**Fig. 15:**
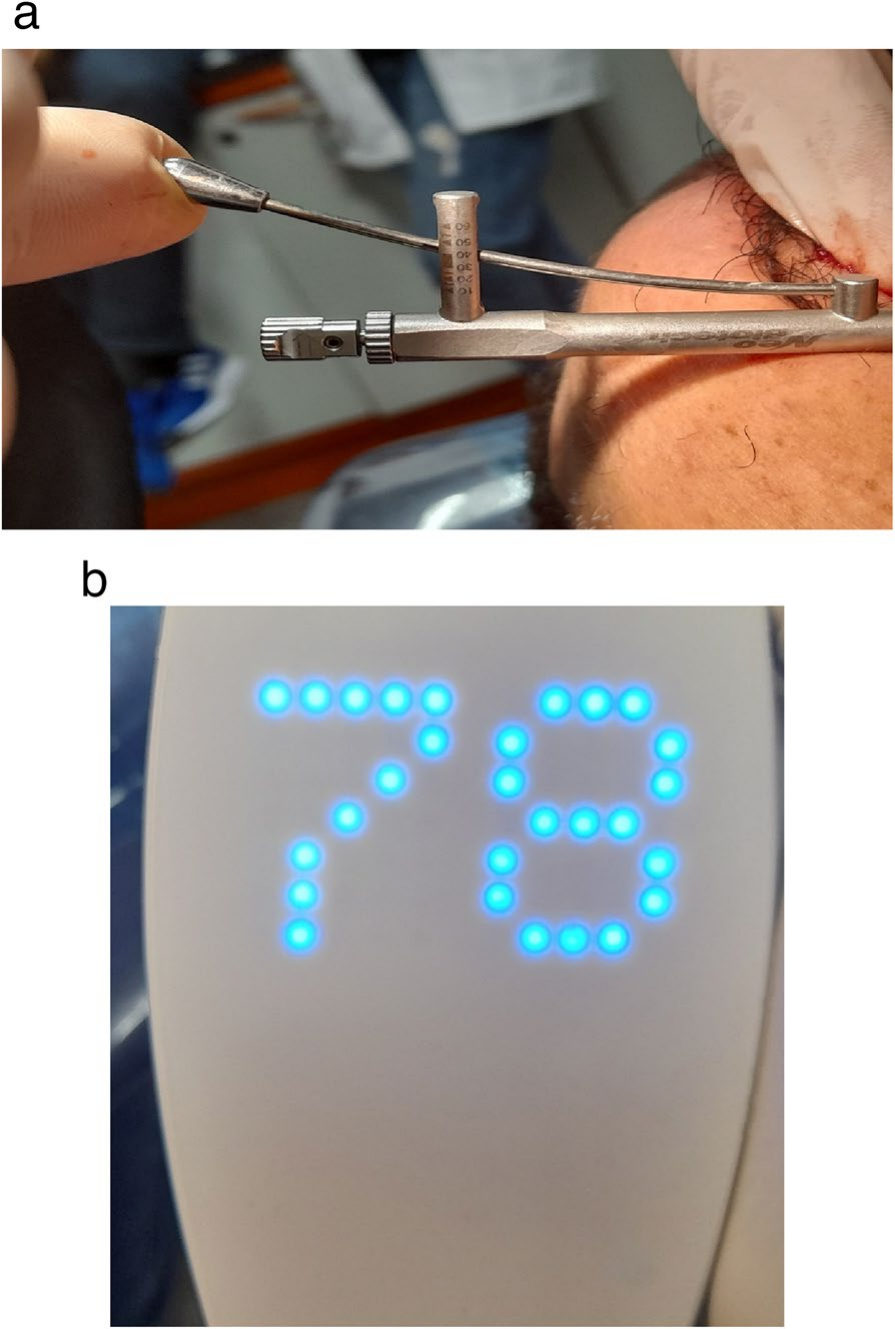
**a** Torque wrench (measuring 45-50 N/cm^2^). **b** ISQ value

**Fig. 16:**
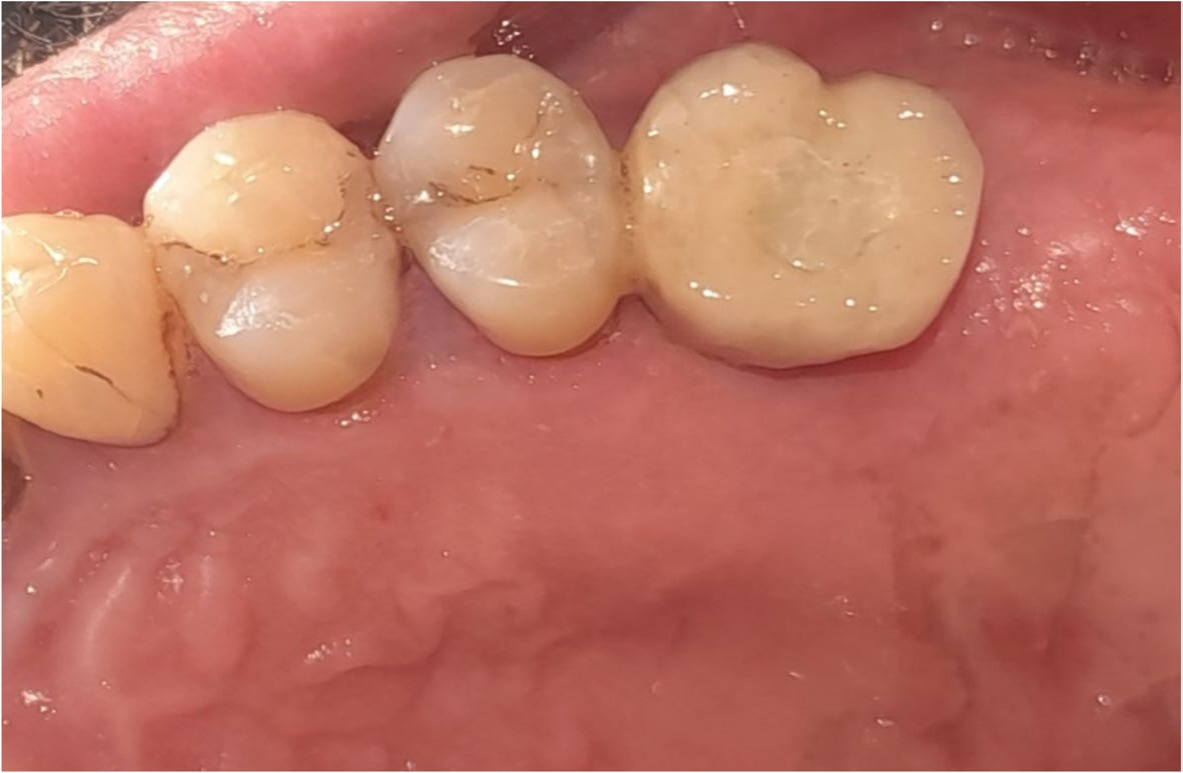
Final screw retained crown after implant osseointegration

In the PISE technique, an ultrasonic piezoelectric device (Acteon. Piezotome®), [38] to which a specialized tip is attached, was used to break the sinus floor. Developed for sinus lift by the crestal approach, the Intralift™ Kit (Figs. 11, 12, 13, 14, 15 and 16) [39] makes it possible to undertake dental non-invasive surgery in full safety. The diamond-coated tips, of increasing diameters (from 1.35mm to 2.80mm), are designed to drill and gradually widen the access canal to the sinus membrane. The sterile spray cools the tips down to avoid any rise in temperature, which could lead to oral tissue damage. The membrane elevation is achieved using the TKW5 (3mm) by means of micro-cavitation. The sinus membrane is gently lifted upward to create a space for bone graft material. The bone graft material is placed in the space created by the lifted sinus membrane using TKW5 tip. Thanks to the ultrasonic frequency modulation, the risk of membrane damage was limited. Moreover, the cavitation effect enables excellent visibility of the operating field. This process was repeated in an incremental fashion to lift the membrane. Once enough space has been created below the antral membrane, implant insertion was done. The insertion torque was measured by torque wrench and ISQ was measured using an Osstell device [37]. Then the suturing was done.

### Postsurgical medication

- Chlorhexidine rinses 0.12 % twice a day for 14 days.
- 1g amoxicillin two times daily for 6 days or 0.5 g azithromycin for patients allergic to penicillin.
- Ibuprofen (400) three times daily unless medically contraindicated.

### Clinical assessment

All implants were evaluated for primary stability once after implant insertion using torque wrench and an Osstell device [37].

### Radiographic assessment

Immediate postoperative CBCTs were taken to all participants to assess vertical bone gain and bone density. *(CBCT i-CAT next-generation scanner (Imaging Sciences International,Hatfield,PA,USA) operating at a tube voltage of 120KVp,tube current of 5mA,voxel size of 0.2mm,field-of-view of 16x6cm ,and scanning time of 26.9seconds)*

**Fig. 17:**
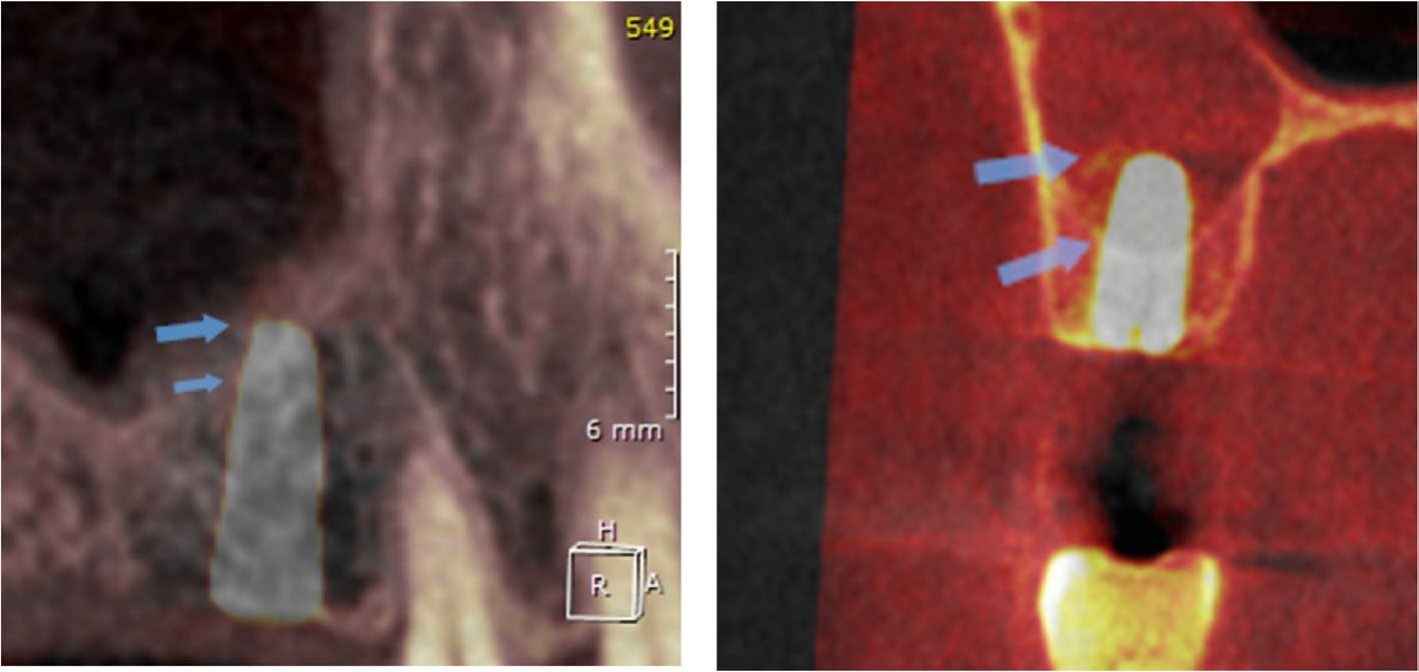
Superimposition of Pre&post-operative CBCT to compare vertical bone gain. Per & post-operative Sinus floor level is marked by the arrows

**Fig. 18:**
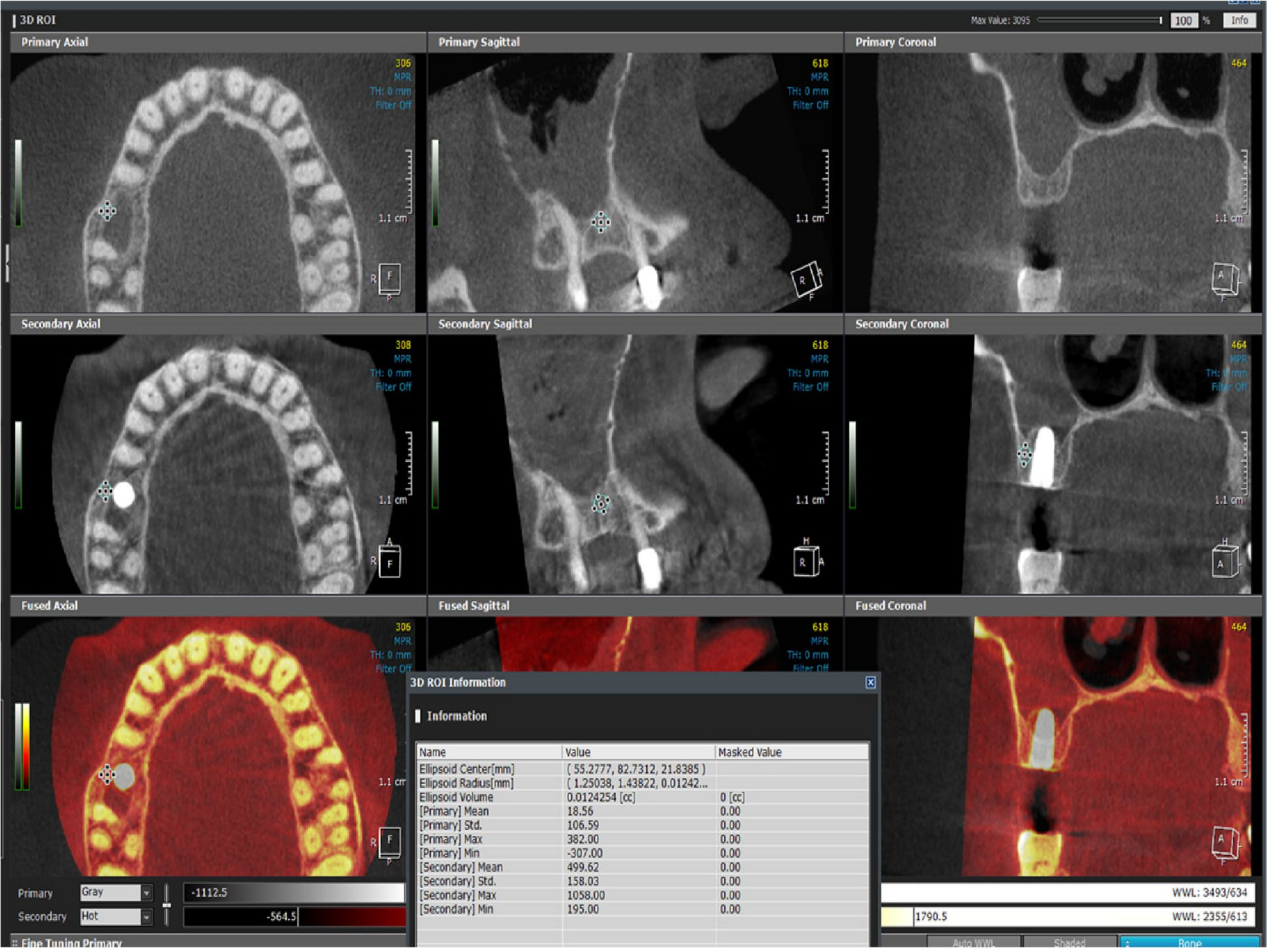
The ROI tool of the software was implemented to measure the bone density buccal and palatal to the implant site in the exact same place and with the exact area on the superimposed per-operative and post-operative CBCTs

OnDemand3D™ imaging software was used to superimpose the per-operative and the immediate post-operative CBCT to help compare the change in the vertical bone height at the implant site and measure the amount of vertical bone gain (Fig. 17), Also, the ROI tool of the software was implemented to measure the bone density buccal and palatal to the implant site in the exact same place and with the exact area on the superimposed per-operative and post-operative CBCTs to help measure and compare the change in bone density after the implementation of both surgical techniques (Fig. 18)

### Assessment of patient and operator satisfaction

- Pain assessment by the aid of the Visual Analogue Scale (VAS).
- A printed questionnaire designed to assess operator subjective satisfaction regarding osseodensification and the PISE techniques was answered postoperatively.
- Another health-related quality of life questionnaire designed to assess patient perception of recovery regarding pain, oral function, general activity, and other symptoms was given and returned from all participants postoperatively at recall visits.

## Statistical methods

Recorded data were analyzed using the statistical package for social sciences, version 23.0 (SPSS Inc., Chicago, Illinois, USA). The quantitative data were presented as mean± standard deviation and ranges when their distribution was parametric (normal) while non-normally distributed variables (non-parametric data) were presented as median with inter-quartile range (IQR). Also, qualitative variables were presented as numbers and percentages. Data were explored for normality using Kolmogorov-Smirnov and Shapiro-Wilk Test.

### The following tests were done

- ▪ *Independent-samples t-test* of significance was used when comparing between two means & *Mann Whitney U test*: for two-group comparisons in non-parametric data.
- ▪ *Paired sample t-test* of significance was used when comparing between related sample & Comparison between two periods for non-parametric data using *Wilcoxon Signed-Rank Sum test*.
- ▪ The Comparison between groups with qualitative data was done by using *Chi-square test* and *Fisher’s exact test* instead of Chi-square test only when the expected count in any cell was less than 5.
- ▪ *The confidence interval was set to 95%* and the margin of error accepted was set to 5%. So, the *p*-value was considered significant as the following:
- ▪ *Probability (P-value)*
  - *P*-value ≤0.05 was considered significant.
  - *P*-value ≤0.001 was considered as highly significant.
  - *P*-value >0.05 was considered insignificant.

## Results

**Fig. 19:**
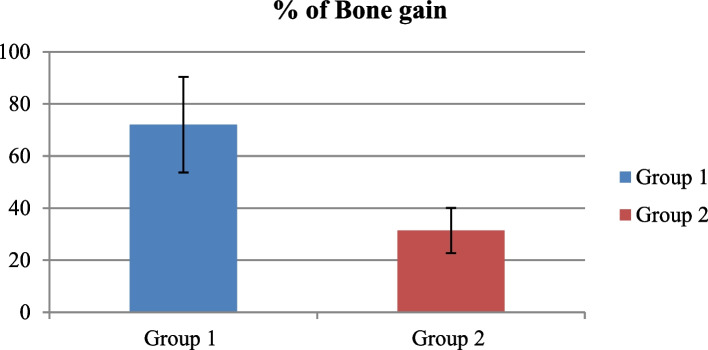
Comparison between Group 1 and Group 2 according to % of Bone gain

**Fig. 20:**
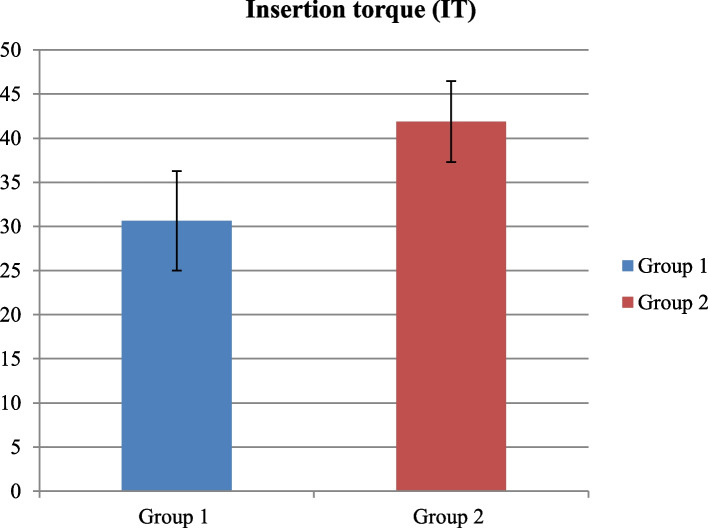
Comparison between Group 1 and Group 2 according to Insertion torque (IT)

**Fig. 21:**
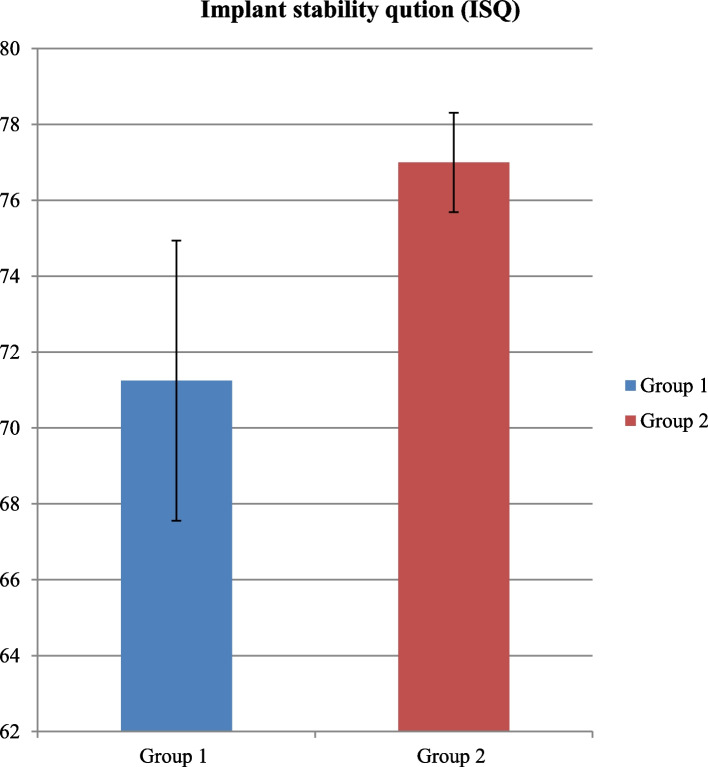
Comparison between Group 1 and Group 2 according to Implant stability quotation (ISQ)

**Fig. 22:**
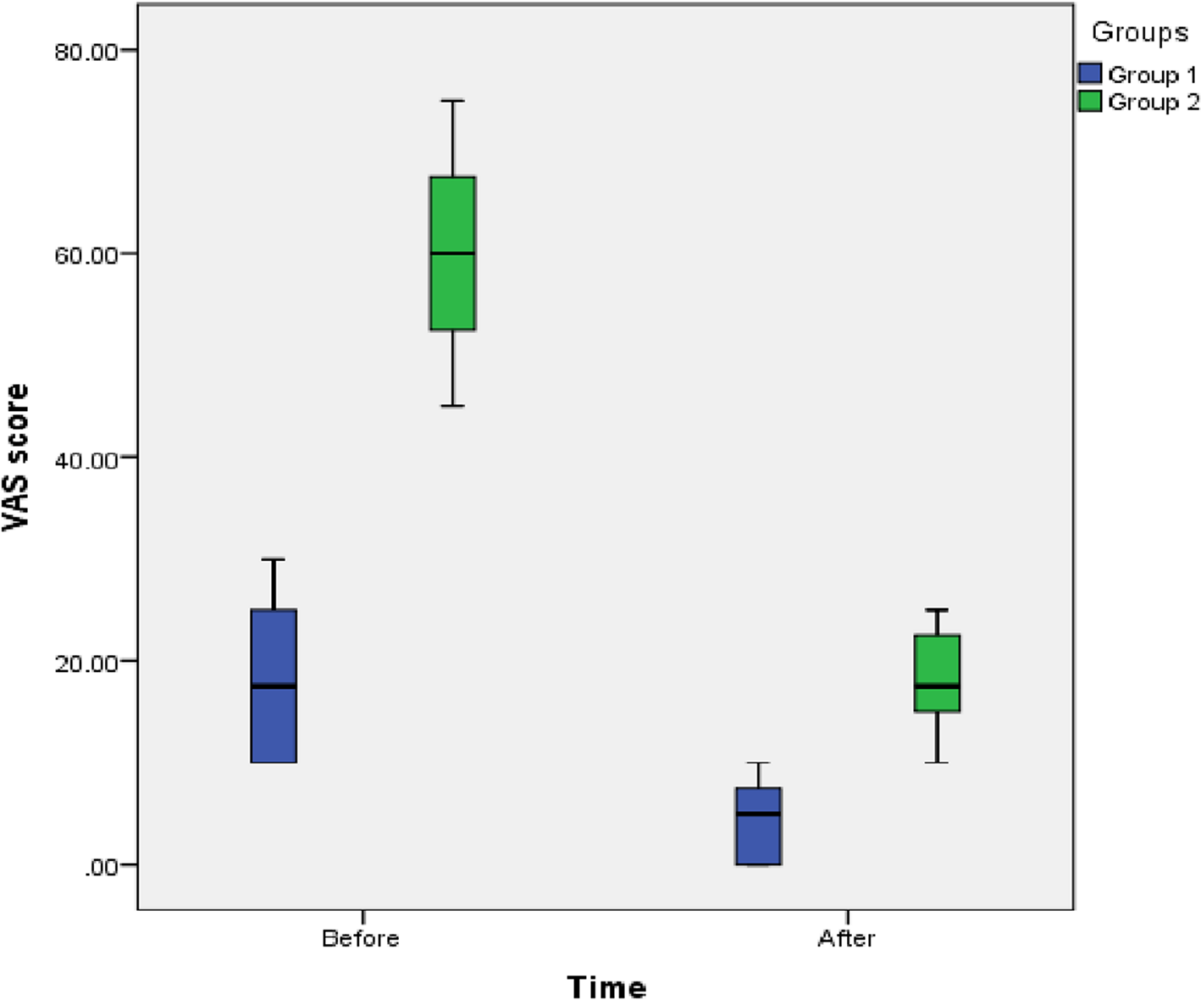
Box plot between Time and VAS score

**Fig. 23:**
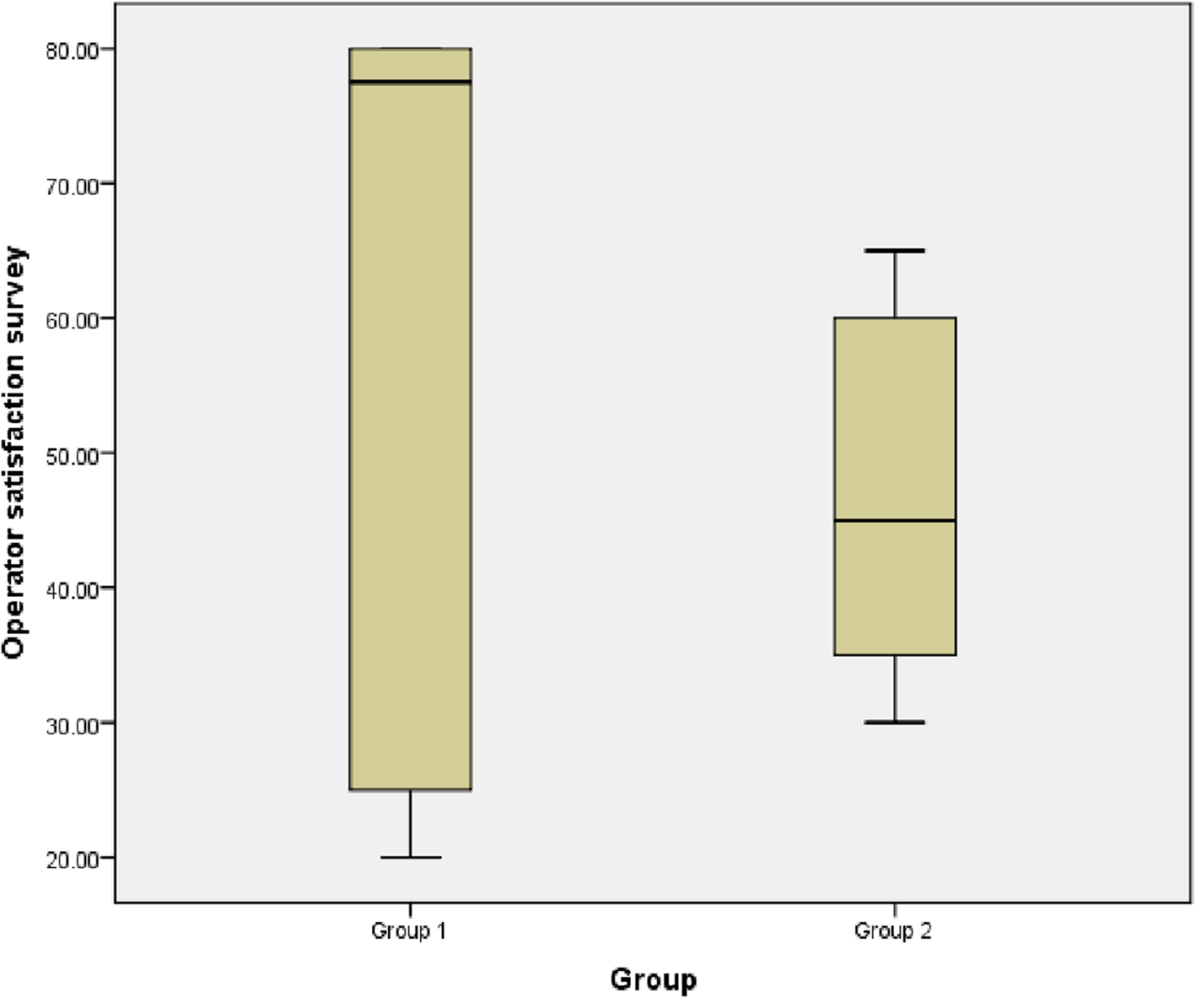
Box plot between Group and Operator satisfaction survey

The study aimed to evaluate the effectiveness and clinical results of osseodensification in comparison to Piezoelectric Internal Sinus Elevation (PISE) Technique in Delayed Implant Placement (Figs. 19, 20, 21, 22 and 23).

### Demographic data

Mean age in group 1 was 40.00±2.67 comparing to group 2 was 41.25±5.95, there is no statistically significant difference between groups, with *p*-value (*p*>0.05); as for the gender, it was female 7 patients (87.5%) and one patient (12.5%) in group 1 comparing to 5 patients (62.5%) were female and 3 patients (37.5%) were male in group 2, but insignificant difference, with *p*-value (*p*>0.05).

### Radiographic analysis

Tables 1 and 2 shows statistically significant higher residual bone height in the two groups in postoperative than preoperative, with *p*-value (*p*<0.05); while there was higher vertical bone gain in group 1 than group 2 and there was a statistically significant difference with *p*-value (*p*<0.05).

**Table 1:** Comparison between Group 1 and Group 2 according to Demographic data

**Demographic data**	**Group 1 (*n*=8)**	**Group 2 (*n*=8)**	**Test value**	***p*-value**
**Age "years"**				
Mean±SD	40.00±2.67	41.25±5.95	-0.542	0.596
Range	38-45	36-48		
**Gender**				
Female	7(87.5%)	5(62.5%)	1.333	0.248
Male	1(12.5%)	3(37.5%)		

Using: t-Independent Sample t-test for Mean±SD;

x^2^: Chi-square test for Number (%) or Fisher’s exact test, when appropriate

*p*-value>0.05 is insignificant; *p-value <0.05 is significant; **p-value <0.001 is highly significant

**Table 2:** Comparison between Group 1 and Group 2 according to Residual bone height (RBH) & Vertical bone gain

**Residual bone height (RBH)**	**Group 1 (*n*=8)**	**Group 2 (*n*=8)**	**Test value#**	***p*-value**
**Preoperative**				
Mean±SD	7.01±1.46	8.50±0.47	-1.954	0.096
Range	5.52-9.22	8-9.24		
**Postoperative**				
Mean±SD	11.64±0.54	11.13±0.83	1.475	0.162
Range	11-12.21	10.14-12.51		
**Bone Gain**	4.64±1.27	2.63±0.69	3.074	0.008*
**% of Bone gain**	72.02±18.35	31.36±8.69	2.889	0.012*
**Paired Sample t-test**	**7.862**	**9.474**		
***p*-value**	0.001*	0.001*		

Using: #t-Independent Sample t-test for Mean±SD;

*p*-value>0.05 is insignificant; *p-value <0.05 is significant

Table 3, shows statistically significant higher bone density in each group in postoperative than preoperative, with *p*-value (*p*<0.05); while there was higher amount of change for bone density in group 1 than group 2, but insignificant difference, with *p*-value (*p*>0.05).

**Table 3:** Comparison between Group 1 and Group 2 according to Bone density

**Bone density**	**Group 1 (*n*=8)**	**Group 2 (*n*=8)**	**Test value**	***p*-value**
**Before procedures**				
Median (IQR)	159.0(140.8-194.6)	193.0(148.1-291.9	-0.898	0.369
Range	72.4_717.4	-4.1_346.9		
**Postoperative**				
Median (IQR)	562.5(407.2-827.8)	604.9(589.2-670.5	-0.800	0.424
Range	407.2_880.2	413.4_670.5		
**Amount of change**	325.9(266.4-448.8)	443.8(297.3-484.9	-1.122	0.266
**% of change**	203.4(189.1-230.3)	168.2(77.8-261.2	-0.158	0.874
**Wilcoxon test**	**2.672**	**2.536**		
***p*-value**	0.010*	0.011*		

Using: #U=Mann-Whitney test for Non-parametric data “Median (IQR)”;

*p*-value>0.05 is insignificant; *p-value <0.05 is significant

### Clinical analysis

There was higher mean value of insertion torque in group 2 was 41.88±4.58 comparing to group 1 was 30.63±5.63, with statistically significant *p*-value (*p*<0.05).

There was higher mean value of implant stability quotation (ISQ) in group 2 was 77.00±1.31 comparing to Group 1 was 71.25±3.69, with statistically significant *p*-value (*p*<0.05) (Tables 4 and 5).

**Table 4:** Comparison between Group 1 and Group 2 according to Insertion torque (IT)

**Insertion torque (IT)**	**Group 1 (*n*=8)**	**Group 2 (*n*=8)**	**Test value**	***p*-value**
Mean±SD	30.63±5.63	41.88±4.58	-4.384	0.001*
Range	25-40	35-45		

Using: t-Independent Sample t-test for Mean±SD;

*p*-value>0.05 is insignificant; *p-value <0.05 is significant

**Table 5:** Comparison between Group 1 and Group 2 according to Implant stability quotation (ISQ)

**Implant stability qution (ISQ)**	**Group 1 (*n*=8)**	**Group 2 (*n*=8)**	**Test value**	***p*-value**
Mean±SD	71.25±3.69	77.00±1.31	-4.150	0.001*
Range	68-78	75-78		

Using: t-Independent Sample t-test for Mean±SD;

*p*-value>0.05 is insignificant; *p-value <0.05 is significant

### Patient satisfaction

Table 6 shows statistically significant lower VAS score in the two groups after one week follow up than on the day of procedure, with *p*-value (*p*<0.05); while there was higher VAS score in group 2 than group 1 and there was a statistically significant difference with *p*-value (*p*<0.05).

**Table 6:** Comparison between Group 1 and Group 2 according to VAS score

**VAS score**	**Group 1 (*n*=8)**	**Group 2 (*n*=8)**	**Test value#**	***p*-value**
**Preoperative**				
Median (IQR)	17.5(10.0-25.0)	60.0(51.3-68.8)	-3.376	0.001*
Range	10-30	45-75		
**Postoperative**				
Median (IQR)	5.0(0.0-8.8)	17.5(15.0-23.8)	-3.300	0.001*
Range	0-10	10-25		
**Reduction of VAS**	12.5	42.5	4.569	0.001*
**Wilcoxon test**	**2.762**	**3.128**		
***p*-value**	0.012*	0.009*		

U=#Mann-Whitney test for Non-parametric data “Median (IQR)”;

*p*-value>0.05 is insignificant; *p-value <0.05 is significant

There was higher operator satisfaction in group 1 was 77.5 (23.8-80.0) comparing to group 2 was 45.0 (33.8-61.3), but insignificant difference, with *p*-value (*p*>0.05) (Table 7).

**Table 7:** Comparison between Group 1 and Group 2 according to Operator satisfaction survey

**Operator satisfaction survey**	**Group 1 (*n*=8)**	**Group 2 (*n*=8)**	**Test value**	***p*-value**
Median (IQR)	77.5(23.8-80.0)	45.0(33.8-61.3)	-0.969	0.332
Range	20-80	30-65		

Using: U=Mann-Whitney test for Non-parametric data “Median (IQR)”;

*p*-value>0.05 is insignificant; *p-value <0.05 is significant

Table 8 shows that the higher frequency of problems opening the mouth in group 2 was 4 patients (50%) comparing to group 1 was 2 patients (25%), also higher frequency of people understand you when you speak in group 1 was 6 patients (75%) comparing to group 1 was 5 patients (62.5%), additionally, there was a higher frequency of anxiety occurs when sutures are removed in group 2 was 3 patients (37.5%) comparing to group 1 was 2 patients (25%), but insignificant difference, with *p*-value (*p*>0.05). As for the problems falling asleep, there was a statistically significant higher in group 2 was 3 patients (37.5%) comparing to group 1 was one patient (12.5%), with p-value (*p=*0.034).

**Table 8:** Comparison between Group 1 and Group 2 according to patient satisfaction survey

**Patient satisfaction survey**	**Group 1**						**Group 2**						**Test value**	***p*-value**
	**Yes**		**No**		**A little**		**Yes**		**No**		**A little**			
	**No.**	**%**	**No.**	**%**	**No.**	**%**	**No.**	**%**	**No.**	**%**	**No.**	**%**		
**Eating ability and diet variation**														
Did you continue with your usual diet?	1	12.5%	6	75.0%	1	12.5%	1	12.5%	6	75.0%	1	12.5%	0.000	1.000
Did you notice any changes in perception of taste?	0	0.0%	7	87.5%	1	12.5%	2	25.0%	4	50.0%	2	25.0%	3.152	0.207
Did you notice any change in chewing efficiency?	1	12.5%	5	62.5%	2	25.0%	3	37.5%	2	25.0%	3	37.5%	2.486	0.288
Did you Have problems opening your mouth?	2	25.0%	5	62.5%	1	12.5%	4	50.0%	1	12.5%	3	37.5%	4.333	0.115
**Speaking ability noticed:**														
Have you noticed any change in voice?	1	12.5%	5	62.5%	2	25.0%	1	12.5%	3	37.5%	4	50.0%	1.167	0.558
Have you noticed any change in your ability to speak?	1	12.5%	7	87.5%	0	0.0%	1	12.5%	4	50.0%	3	37.5%	3.818	0.148
When you talk with people, do they understand you?	6	75.0%	0	0.0%	2	25.0%	5	62.5%	1	12.5%	2	25.0%	1.091	0.579
**Sleep impairment:**														
Have you had problems falling asleep?	1	12.5%	7	87.5%	0	0.0%	3	37.5%	2	25.0%	3	37.5%	6.778	0.034*
Have you experienced interruptions in sleep?	0	0.0%	5	62.5%	3	37.5%	2	25.0%	4	50.0%	2	25.0%	2.311	2.311
Have you felt drowsy?	0	0.0%	6	75.0%	2	25.0%	1	12.5%	6	75.0%	1	12.5%	1.333	0.513
**Pain and discomfort at suture removal**														
Has the removal of sutures been uncomfortable?	1	12.5%	5	62.5%	2	25.0%	1	12.5%	5	62.5%	2	25.0%	0.000	1.000
Has the appointment for suture removal caused you anxiety?	2	25.0%	4	50.0%	2	25.0%	3	37.5%	2	25.0%	3	37.5%	1.067	0.586

x2: Chi-square test for Number (%) or Fisher’s exact test, when appropriate

*p*-value>0.05 is insignificant; *p-value <0.05 is significant; **p-value <0.001 is highly significant

## Discussion

Tooth loss can lead to maxillary sinus pneumatization, which subsequently causes the fusion of the alveolar crest and the sinus floor. This often results in inadequate vertical bone volume and height [40]. To overcome this constraint, several procedures have been developed: including tilted implants [41, 42], zygomatic implants [43], and short implants [44].

The usage of bone substitutes to improve vertical bone height in the floor of the maxillary sinus was first described by Boyne et al., [45]. It is becoming more common to repair vertically resorbed ridges before implant placement to optimize implant fixation and future osseointegration [46, 47].

To ensure successful implant therapy, various sinus floor lifting techniques have been tried to increase the availability of bone in this location [48].

Tatum proposed the procedure in 1986, which involved surgical access gained to the sinus by entering the lateral wall of the maxillary zygomatic buttress followed by insertion of bone grafting substance [49]. One of the common consequences of this approach was perforated maxillary sinus membrane with rate up to 35% of surgeries [50, 51].

Summers later improved the procedure by using compressive osteotomes and hand devices to lift the sinus membrane utilizing an alveolar approach to increase the alveolar ridge height [49, 52].

Even though this approach is more widely utilized and less intrusive than the lateral one, it has been found to have significant drawbacks. For example, the increased bone volume is restricted, Benign paroxysmal positional vertigo, and there is no direct visual control over the reliability of the Schneiderian membrane [53, 54].

The direct sinus lift utilizing a balloon through a lateral window wall was described by Muronoi et al., in 2003 [55], and Soltanet al., in 2005 [56]. The membrane is gently detached with a latex balloon inflated with saline solution and inserted via a hole through the lateral wall of the sinus.

In 2011, Kfir et al., [57] demonstrated a crestal sinus elevation utilizing the inflating balloon procedure, with placement of dental implants and bone grafts simultaneously in the same surgical step It is, nonetheless, technically challenging, and cost effective [48].

In 2001, Vercellotti introduced an advanced sinus elevation technique using an ultrasonic surgical method called piezoelectric bony window osteotomy for maxillary sinus surgery. This approach utilizes a physiological solution subjected to piezoelectric cavitation and piezoelectric elevators to lift the Schneiderian membrane from the sinus floor. Due to its operational frequency of 25-29 kHz, piezoelectric internal sinus elevation provides excellent tactile feedback. [18, 48] and limited bone cutting of just mineralized structures [58, 59].

Unlike previous transcrestal sinus lift procedures, this one is non-invasive. It is not dependable on compaction of the bone to lift the sinus membrane. The technique utilizing ultrasonic vibration together with hydraulic pressure to uplift the sinus membrane aided with vigorous irrigation may lead to breakage of the sinus floor [60].

It has been reported that the hydrodynamic pressure applied to the membrane is uniformly dispersed because of its centrifugal direction, which causes the Schneiderian membrane to gradually detach [48, 61]. Pressure applied to the membrane only placed to its top when employing osteotomes lift procedure followed by hand instruments, and while pressing upwards the whole membrane is subjected to ripping stresses however it is not sufficiently raised in comparison to intra-lift sinus procedure [48, 59–61]. Less trauma, reduced operation time, and a diminished risk of postoperative perforation and morbidity are all advantages of the transrectal sinus floor elevation procedure in comparison to the lateral sinus floor elevation procedure [58, 59, 61].

A novel osseodensification approach was introduced in 2016 by Huwais and Meyer [19].

The use of Densah burs for maxillary sinus lifting was first introduced by Huwais and Meyer in 2018 [62] utilizing the advantages of the osseodensification approach for elevation of the maxillary sinus floor. The idea of compaction autografting is supported by the design of Densah burs with specially tapered geometry and specially designed flutes to compact the bone on its walls and apex [62].

The concept involves a unique flute design in a densifying, non-cutting mode that rotates counterclockwise. When combined with irrigation, this setup generates a hydraulic wave at the bur’s apex, which pushes the sinus membrane upward. The presence of grafting material enhances this effect, leading to the elevation of the Schneiderian membrane with a minimized risk of perforation [62]. So this approach is suggested to provide a safe technique for maxillary sinus lifting with limited complications as in osteotome or lateral approach, less perforation and less invasiveness [62].

Since the introduction of Densah burs, a few studies have assessed their effectiveness and patient experiences. Osseodensification can prepare the implant site and elevate the sinus membrane with a low risk of perforation and minimal postoperative complications. It also supports autogenous bone grafting, enhancing implant stability. These benefits stem from a combination of hydrodynamic wave action and hydraulic compression [19].

Up till now there are limited studies to evaluate the transcrestal sinus lift and simultaneous implant placement using osseodensification and piezoelectric internal sinus elevation (PISE) technique Therefore, this study was designed to evaluate the effectiveness and clinical results of osseodensification in comparison to piezoelectric internal sinus elevation (PISE) technique in delayed implant placement with placement of sticky bone as bone grafting material since it allows ease of handling of the graft material Furthermore, promoting healing by the significant release of cytokines and autologous growth factors [33, 63, 64].

In this study participants were divided into two groups:

Group 1 (*n*=8): Osseodensification Group 2 (*n*=8): Piezoelectric Internal Sinus Elevation (PISE) with no significant differences concerning mean age or gender of the patients.

And both surgical techniques proved effective in sinus elevation with statistically significant higher residual bone height in the two groups in postoperative than perioperative with *p*-value (*p*<0.05) and that confirms the results of similar studies [35, 65–67].

While the inter-group comparison of the vertical bone gain showed a statistically significant higher bone gain in the osseodensification group than piezoelectric group and that contradicts to the results of similar studies [35, 65–67]. And this contradiction can be attributed to the difference in the scientific background of the surgical technique itself as in osseodensification the special design of flutes in the densifying non-cutting mood with counter clockwise motion and presence of irrigation cause a hydraulic wave at the apex of the bur, this wave cause pushing of the sinus membrane upward, also in presence of grafting material cause the same effect and subsequent elevation of the Schneiderian membrane with limited risk of perforation as mentioned by [62, 68] so it localize the elevation effect to the implant site alone while In case of piezoelectric sinus elevation.

It is not dependable on compaction of the bone to lift the sinus membrane. The technique utilizing ultrasonic vibration together with hydraulic pressure to uplift the sinus membrane aided with vigorous irrigation may lead to breakage of the sinus floor [60].

It has been reported that the hydrodynamic pressure applied to the membrane is uniformly dispersed because of its centrifugal direction, which causes the Schneiderian membrane to gradually detach [48, 61].

Both surgical techniques showed a statistically significant higher bone density postoperative than perioperative that confirms the results of similar studies [35, 65–67]. while there was higher amount of change for bone density in osseodensification group than piezoelectric group, but insignificant difference, with *p*-value (*p*>0.05). and that confirms the results of similar studies [35, 65–67]and this can be attributed to the ability of osseodensification drills to densify and compact bone in the osteotomy site.

Concerning implant primary stability there was a statistically significant higher mean value of insertion torque in piezoelectric group comparing to osseodensification group and, a statistically significant higher ***ISQ*** value in piezoelectric group comparing to osseodensification group and that contradicts the results of [35, 65–67].

And this contradiction can be attributed to the undersized osteotomy used with the piezoelectric group of our study because as mentioned TWK5 measures only 3mm in diameter and the inserted implant was 4mm-4.5mm diameter and the shaping drills of the implant was not used to avoid any confounding factors or any risk of sinus perforation while in the osseodensifiction group the final drill used measures 3.5mm and it was used multiple times in and out of the osteotomy site in order to elevate the sinus membrane and compact the graft incrementally 1mm at time resulting in an exact size osteotomy compatible to 4mm-4.5mm implant diameter , despite the contradiction on the statistical analysis results between our study and the perivenous mentioned studies [35, 65–67] the exact insertion torque readings and the ISQ values of the osseodensifiction group of our study confirms to that found in the same groups in [35, 65, 67].

Concerning the patient satisfaction of both techniques our studied showed low VAS scores for both techniques and that confirms to the results of previous studies by [35, 65]. while there was higher VAS score in piezoelectric group than osseodensifiction group and there was a statistically significant difference with *p*-value (*p*<0.05), and this can be attributed to the effect of the hydrodynamic pressure of the irrigation solution of the piezoelectric sinus elevation technique as it was in direct contact with the sinus membrane causing slight pain and edema post-operatively while in the osseodensifiction group the burs was always in contact with the sinus floor or the graft material and there was no direct contact with the sinus membrane explaining lower levels of pain and edema post-operatively [35].

## Conclusion

Considering the limitations of the current clinical trial both techniques of transcrestal sinus lift were successful in sinus floor elevation and provided excellent clinical results.

The osseodensification demonstrated greater bone gain, better bone density, and shorter surgical duration. Moreover, the osseodensification technique shows a reliable method to enhance rapid healing and more patient satisfaction, while the piezoelectric sinus elevation technique should better primary stability for implants on the day of surgery.

### Limitations and suggestions

1. Greater sample size is recommended understand the critical relationship among variables and their significance.
2. A longer follow-up phase of up to 1 or 2 years is required to determine the bone density around the implant, which is an important outcome to consider.

## Data Availability

No datasets were generated or analysed during the current study.

## Abbreviations

β-TCP: Beta-Tri-calcium phosphate
BMPs: Bone morphogenetic proteins
CBCT: Cone-beam computed tomography
CGF: Concentrated growth factors
EGF: Epidermal growth factor
FGF: Fibroblast growth factor
HU: Hounsfield Units
IGF: Insulin-like growth factor
I-PRF: Injectable platelet-rich fibrin
IQR: Inter-quartile range
ISQ: Implant Stability Quotient
LWT: Lateral Window Technique
PDGF: Platelet-derived growth factor
PISE: Piezoelectric Internal Sinus Elevation
PRP: Platelet Rich Plasma
ROI: Region of Interest
SFE: Sinus floor elevation Technique
TGF-β: Transforming growth factor-β
VAS: Visual Analogue Scale
VEGF: Vascular endothelial growth factor
